# High percentage of abnormal findings on TVU needs further discussion

**DOI:** 10.1038/sj.bjc.6603408

**Published:** 2006-10-17

**Authors:** M J E Mourits, N M van der Velde, H J G Arts, G H de Bock

**Affiliations:** 1 Department of Obstetrics and Gynaecology, University Medical Centre Groningen, University of Groningen, PO Box 30.001, Hanzeplein 1, Groningen 9700 RB, The Netherlands; 2 Department of Epidemiology, University Medical Centre Groningen, University of Groningen, Groningen, The Netherlands

**Sir**,

With great interest, we read the publication ‘Surveillance of women at high risk for hereditary ovarian cancer is inefficient’ published by Oei *et al* (2006) in your journal. The authors evaluated the efficacy of 10-year gynaecological surveillance in 512 women at risk for hereditary ovarian cancer in a tertiary referral centre. This gynaecological surveillance included annual pelvic examination, transvaginal ultrasound (TVU) and serum CA125 measurements. In case of abnormal findings in at least one of the surveillance tests, revision of the abnormal test after 3–6 months was advised. A total of 1621 surveillance tests were performed. In 317 contacts (21%), TVU showed abnormal findings. Of these, 295 (93%) were not present during repeat TVU after 3–6 months. Because only two cases of ovarian cancer were diagnosed during this annual surveillance, it can be calculated that the highest estimate for a positive predictive value of an abnormal TVU is 0.6%.

This figure of 21% abnormal findings on TVU needs further discussion than the authors give in this paper. The criteria in the method section of this study, according to which TVU was considered as abnormal were: multiple cysts, cysts with thick septa, papillary projection, irregular patterns or a variety in sonolucency. In the discussion section, the authors explain their high percentage of false-positive findings on TVU with the observation that the majority of their patients were premenopausal and that a variety of benign conditions (e.g. follicle cysts) have contributed to the high incidence of normalising abnormalities. It is remarkable that the list of criteria used in this study according to which TVU was considered abnormal, does not include the presence and/or size of monolocular cysts. According to the Sassone criteria, only cysts of more than 6 cm need further investigation in premenopausal women (Sassone *et al*, 1991). A second point of attention is that it is not mentioned whether the authors found any adverse effects in this group of patients who had to wait for 3–6 months before they could be reassured by a repeat TVU. It is known from literature that ovarian cancer screening in high-risk women can have a great impact on the anxiety score, especially of premenopausal women (Hensley *et al*, 2003).

We agree with the authors that surveillance is highly ineffective to detect early ovarian (tubal) cancer in high-risk women. It can be questioned whether their suggestion to conduct a study with a larger group of BRCA mutation carriers with a longer follow-up is in line with this conclusion. However, if they need more definitive conclusions, as they suggested, they should define their TVU criteria more precisely in order to avoid this high percentage of false-positive TVU findings and the high loss of quality of life in women with a false-positive TVU finding.
